# Engineering Clock Transitions in Molecular Lanthanide Complexes

**DOI:** 10.1021/jacs.3c09353

**Published:** 2024-04-15

**Authors:** Robert Stewart, Angelos B. Canaj, Shuanglong Liu, Emma Regincós Martí, Anna Celmina, Gary Nichol, Hai-Ping Cheng, Mark Murrie, Stephen Hill

**Affiliations:** † National High Magnetic Field Laboratory, Florida State University, Tallahassee, Florida 32310, United States; ‡ Department of Physics, Florida State University, Tallahassee, Florida 32306, United States; § Center for Molecular Magnetic Quantum Materials, University of Florida, Gainesville, Florida 32611, United States; ∥ School of Chemistry, University of Glasgow, University Avenue, Glasgow G12 8QQ, U.K.; ⊥ Department of Physics, Northeastern University, Boston, Massachusetts 02115, United States; # EastCHEM School of Chemistry, The University of Edinburgh, David Brewster Road, Edinburgh EH9 3FJ, Scotland, U.K.

## Abstract

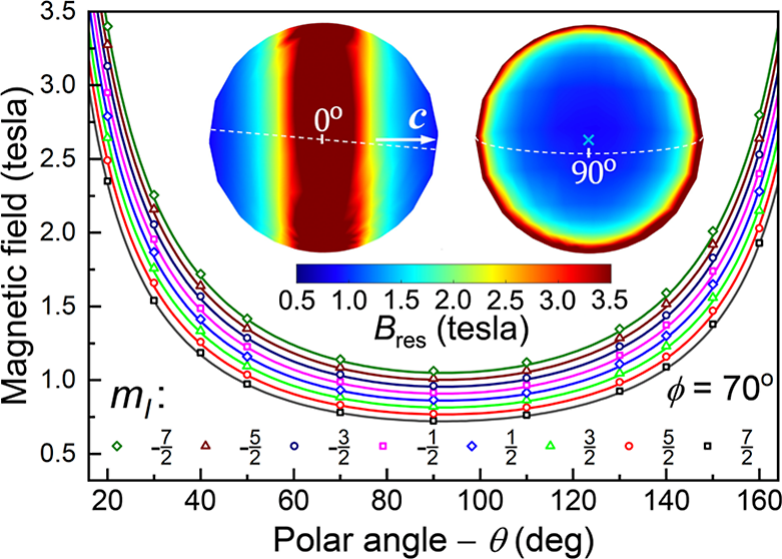

Molecular lanthanide (Ln) complexes are promising candidates for the development of next-generation quantum technologies. High-symmetry structures incorporating integer spin Ln ions can give rise to well-isolated crystal field quasi-doublet ground states, i.e., quantum two-level systems that may serve as the basis for magnetic qubits. Recent work has shown that symmetry lowering of the coordination environment around the Ln ion can produce an avoided crossing or clock transition within the ground doublet, leading to significantly enhanced coherence. Here, we employ single-crystal high-frequency electron paramagnetic resonance spectroscopy and high-level ab initio calculations to carry out a detailed investigation of the nine-coordinate complexes, [Ho^III^L_1_L_2_], where L_1_ = 1,4,7,10-tetrakis(2-pyridylmethyl)-1,4,7,10-tetraaza-cyclododecane and L_2_ = F^–^ (**1**) or [MeCN]^0^ (**2**). The pseudo-4-fold symmetry imposed by the neutral organic ligand scaffold (L_1_) and the apical anionic fluoride ion generates a strong axial anisotropy with an *m*_*J*_ = ±8 ground-state quasi-doublet in **1**, where *m*_*J*_ denotes the projection of the *J* = 8 spin–orbital moment onto the ∼*C*_4_ axis. Meanwhile, off-diagonal crystal field interactions give rise to a giant 116.4 ± 1.0 GHz clock transition within this doublet. We then demonstrate targeted crystal field engineering of the clock transition by replacing F^–^ with neutral MeCN (**2**), resulting in an increase in the clock transition frequency by a factor of 2.2. The experimental results are in broad agreement with quantum chemical calculations. This tunability is highly desirable because decoherence caused by second-order sensitivity to magnetic noise scales inversely with the clock transition frequency.

## 1 Introduction

The superiority of quantum computers for performing certain computational tasks has been well established at the theoretical level,^1,2^ while practical devices are getting ever closer to attaining quantum advantage.^3,4^ However, many challenges remain before the full potential of quantum information science can be unleashed. Foremost among these challenges is scalability, whereby large numbers of addressable quantum bits, or qubits, can be integrated into complex circuitry capable of implementing useful quantum algorithms with embedded quantum error correction.^5,6^ Among the many qubit platforms under consideration^7^ (e.g., solid state defects,^8^ quantum dots,^9^ photons,^3^ trapped atoms/ions,^10,11^ and superconducting circuits^4,12^), electron and nuclear spins in molecules are gaining interest.^13−15^ Molecular spins possess discrete energy levels, while the associated quantum states can be tuned and coherently manipulated using external electromagnetic fields.^16,17^ Crucially, chemistry-inspired supramolecular or self-assembly approaches are well-suited to tackling the issue of scalability.^18−20^

Although several magnetic molecules have been shown to possess excellent coherence properties, this invariably requires extreme dilution in diamagnetic host matrices to minimize potentially long-range spin–spin dipolar interactions that represent a stubborn source of noise (i.e., decoherence);^21^ in some cases, this has even involved the exclusion of most or all magnetic nuclear isotopes.^22,23^ However, there is a fundamental contradiction between such approaches and the aforementioned advantages associated with the bottom-up chemical synthesis of scalable multiqubit assemblies.^16^ That is, dilution would prohibit controlled through-bond interactions between qubits that are essential to the operation of quantum gates,^24^ while exclusion of magnetic nuclei vastly restrict the chemical toolbox that can be employed. To this end, there is an urgent need to develop molecular spin qubits with built-in protection against unwanted sources of magnetic noise. Recent work has shown that this can be achieved by exploiting avoided Zeeman level crossings^25,26^—so-called spin clock transitions (SCTs)—where the dependence of the qubit transition frequency (*f*) on magnetic field (*B*_0_) is suppressed to the first order, i.e., d*f*/d*B*_0_ = 0, thus generating an insensitivity to local magnetic field fluctuations (noise). In particular, the spin states associated with lanthanide ions have proven to be excellent targets.^26−29^ For example, non-Kramers ions with integer spin–orbital momentum, *J*, host tunable SCTs dictated by the local coordination geometry, i.e., the crystal field (CF).^26−32^ Meanwhile, chemical control of the degree of s-orbital mixing into the relevant spin-bearing orbital enables tuning of hyperfine SCTs associated with half-integer angular momentum Kramers ions.^28,33^

In the first molecular SCT example, [Ho(W_5_O_18_)_2_]^9–^, the Ho^III^ center resides in a slightly distorted square-antiprismatic coordination environment with approximate *D*_4*d*_ symmetry.^26,34^ The action of the pseudo-axial CF on the aspherical f-electron density lifts the degeneracy associated with the corresponding *J* = 8 spin–orbital moment. However, subtleties of the Hund’s rule 4f^10^ electronic configuration (possessing both oblate and prolate character)^35,36^ coupled with the disposition of the coordinating oxygens,^30^ which are oriented close to the magic angle (54.7°) relative to the pseudo-*C*_4_ axis, result in stabilization of the *m*_*J*_ = ±4 (projection of *J*) quasi-doublet ground state as opposed to the maximal *m*_*J*_ = ±8 projection.^37^ A pure CF clock-transition would be strictly forbidden for a 4f ion in a hypothetical *D*_4*d*_ coordination geometry, i.e., the *m*_*J*_ = ±4 Zeeman levels would have exact degeneracies at their crossing points. This is due to the time-reversal invariance of the spin–orbit coupling (SOC) interaction that results in a spin Hamiltonian of higher (8-fold rotational) symmetry in comparison to the corresponding molecule (4-fold rotational symmetry in the ideal *D*_4*d*_ case);^38^ this degeneracy would result from restrictions on the allowed off-diagonal 4f CF operators that are restricted to sixth order (ignoring any covalency effects), therefore precluding 8-fold rotational symmetries. Hence, the spin Hamiltonian would have a cylindrical symmetry (*C*_∞_ point group) in this case. However, an exact *D*_4*d*_ molecular geometry is incompatible with any of the 32 crystallographic point groups.^30^ Therefore, minor distortions away from ideal *D*_4*d*_ symmetry in the [Ho(W_5_O_18_)_2_]^9–^ example^34^ give rise to SCTs with corresponding avoided crossing gaps, Δ/*h* = 9.2 GHz (≈0.3 cm^–1^).^26^ Consequently, the magnitudes of these gaps do not so much reflect any tunable property of the ligand but are instead related to unpredictable crystal-packing forces associated with the low-symmetry *P*1® space group.

In this study, we demonstrate that by moving to more tunable complexes, where the lanthanide ion (also Ho^III^ in this case) is encapsulated within a cage-like octadentate ligand scaffold with an open (ninth) axial coordinate site, it is possible to chemically engineer the ground state electronic configuration (*m*_*J*_ = ±8 versus ±4) and tune the corresponding SCT frequencies over a wide range through variation of the axial ligand. Crucially, the encapsulating nature of the ligand, where it wraps around the lanthanide ion, precludes a rotoinversion axis of symmetry, resulting in a pseudo-*C*_4*v*_ coordination geometry. Consequently, the molecular CF interaction naturally generates spin Hamiltonian terms that give rise to sizable SCTs of up to ∼250 GHz, regardless of crystal symmetry. This tunability is highly desirable because second-order sensitivity to magnetic noise scales inversely with SCT frequency.^29^

## 2 Experimental and Computational Details

**Figure 1:**
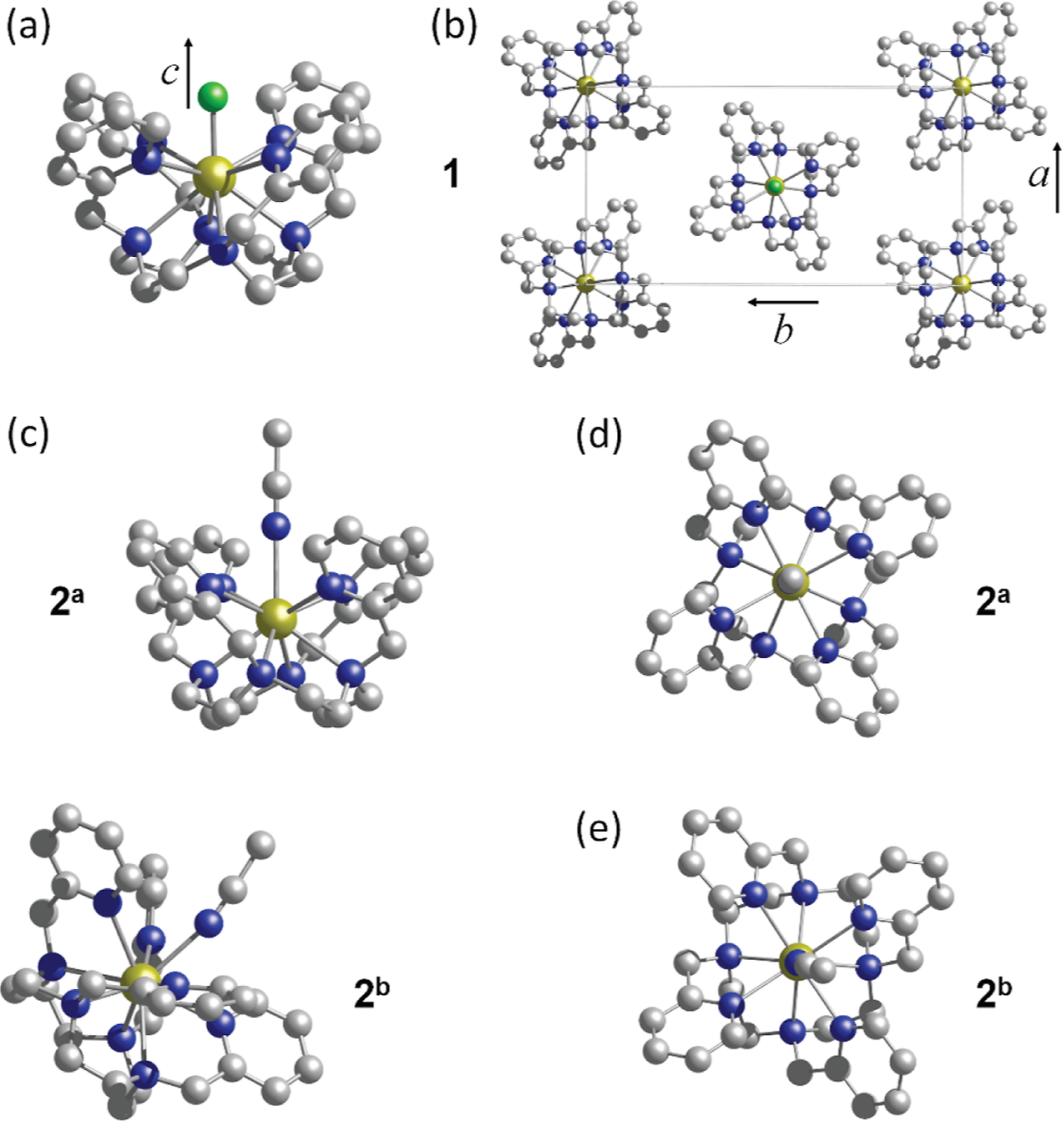
(a) Molecular structure of **1** viewed along the *a*-axis and (b) crystal-packing viewed along the *C*_2_-axis (∥ *c*-axis), displaying the two magnetically equivalent sets of molecules related by 2_1_ screw operations. (c) Asymmetric unit of **2** displaying the magnetically inequivalent molecules, **2**^**a**^ and **2**^**b**^, also shown, respectively, in (d,e), as viewed along their pseudo-*C*_4_ axes. Carbon (gray), holmium (gold), fluorine (green), nitrogen (blue); hydrogens as well as the counterions and solvent molecules have been omitted for clarity.

High-field EPR (HFEPR) studies were performed on Ho^III^ members of the 9-coordinate lanthanide complexes, [Ln^III^LF](CF_3_SO_3_)_2_·H_2_O (**1**) and [Ln^III^L(MeCN)](CF_3_SO_3_)_3_·0.5MeCN (**2**), where the neutral ligand L = 1,4,7,10-tetrakis(2-pyridylmethyl)-1,4,7,10-tetraaza-cyclododecane;^39^ the molecular structures are shown in Figure 1. Compound **1** was synthesized according to the procedure described previously,^40^ while compound **2** was prepared by adaptation of a previous method.^41^

**Figure 2:**
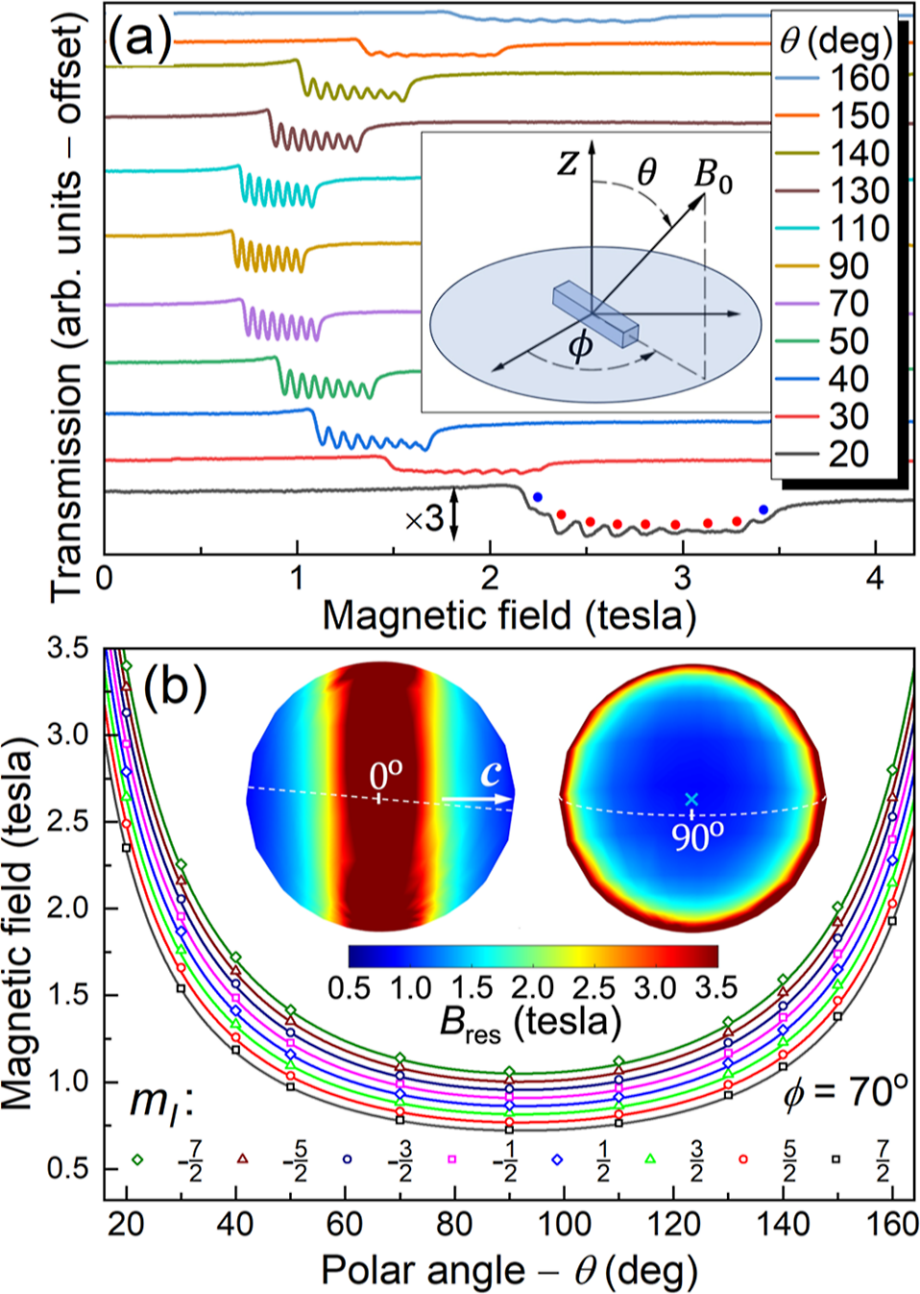
(a) 259 GHz HFEPR spectra of **1** recorded at 2 K as a function of the polar angle, θ (see legend), for the ϕ = 70° plane of rotation (see inset defining the experimental coordinates relative to the rod-shaped sample resting horizontally on the circular end-plate of the cylindrical cavity); the θ = 20° spectrum has been expanded ×3 vertically, and the resonances are indicated with red and blue dots (see the main text for a further discussion). (b) Positions in magnetic field of the eight hyperfine transitions (dips in transmission) observed in (a) for each value of θ; the associated *m*_*I*_ values are given in the legend and the solid curves are simulations based on the spin-Hamiltonian parameters given in Table 1 (see below). The inset displays spherical false color plots of the pure electronic resonance field, *B*_res_(θ,ϕ), from two orthogonal perspectives (see the text for an explanation); the crystal *c*-axis is shown (also by ×) and the dashed lines denote the ϕ = 70° plane of rotation, with θ = 0 and 90° indicated as points of reference.

Continuous-wave HFEPR measurements were performed on single crystals of **1** and **2** using a 9-5-1 T vector magnet (Cryogenic Ltd., UK), with in situ two-axis rotation capabilities;^42,43^ all experiments were performed at 2 K using the variable-flow ^4^He cryostat associated with the vector magnet. Multifrequency spectra were recorded using a cavity perturbation technique with a Millimeter-wave Vector Network Analyzer (AB Millimetre, France) serving as a microwave source and detector.^44,45^ Resonant microwave absorption is observed as dips in the transmission through the cavity. A rod-shaped crystal of **1** with approximate dimensions 0.5 × 0.5 × 3 mm^3^ was mounted horizontally on the base plate of a cylindrical resonator. Angle-dependent studies were then conducted by adjusting the polar angle, θ, of the applied field, *B*_0_, from 0 to 180°, using the 9-5 T coils of the magnet (maximum vector field of 4.5 T), where θ = 0° is coincident with the vertical cylindrical (*z*-) axis of the resonator. Meanwhile, the azimuthal angle ϕ was varied in 10° increments from 0 to 180° by physically rotating the resonator about its cylindrical axis (see inset to Figure 2a). Field sweeps from 0 to 4.5 T were recorded at each (θ,ϕ) orientation at a single frequency of 259 GHz. The axial symmetry direction (quantization axis) of the crystal was then determined after careful study of the angle-dependent spectra, and a frequency dependence was subsequently performed with the applied field parallel to the symmetry axis over the range from 100–320 GHz.

A thin plate-like crystal of **2** was placed with the large face perpendicular to the cylindrical axis of the resonator (θ = 0°). Angle-dependent measurements for **2** were greatly complicated by the low-symmetry space group, with two inequivalent molecules in the asymmetric unit (vide infra), as well as some disorder in the structure that contributes to increased resonance line widths.^46−49^ Therefore, the main conclusions of this study are based on frequency dependence measurements performed in the range from 100–309 GHz, which enable an estimate of the zero-field SCT gap, Δ. All spectral simulations were performed using EasySpin.^50^

Complete active space self-consistent field (CASSCF) calculations^51,52^ were performed using the ORCA package^53^ to understand the electronic structure of the molecules as well as the EPR spectra. The scalar relativistic second order Douglas–Kroll–Hess (DKH) Hamiltonian^54,55^ was adopted for describing the heavy element Ho. The active space was chosen as 10 electrons in 7 orbitals. Dynamical correlation beyond the active space was added through the second-order N-electron valence state perturbation theory (NEVPT2).^56−59^ The approach of domain-based local pair natural orbitals was applied to speed up the NEVPT2 calculations.^60^ The resolution of the identity approximation was not invoked in the CASSCF calculations. The SARC2-DKH-QZVP basis set^61^ was chosen for the Ho atom, and the DKH-DEF2-TZVP basis set^62,63^ was used for all the other atoms. SOC was included via the quasi-degenerate perturbation theory, also known as the state interaction method,^64,65^ which was based on the 35 lowest-energy spin-2 roots, the 105 lowest-energy spin-1 roots, and the 21 lowest-energy spin-0 roots (511 states in total after spin splitting). The second-order DKH transformation was applied for the one-electron part of the SOC operator.^66,67^ The two-electron part of the SOC operator was treated by the spin–orbit mean field approximation.^68^ The SINGLE_ANISO program^69,70^ was used to calculate the CF parameters and analyze the state composition.

## 3 Results and Discussion

The high-symmetry [Ho^III^LF]^2+^ complex in **1** contains a {Ho^III^N_8_} cage with a distorted square antiprismatic geometry. However, as seen in Figure 1a, the Ho^III^ ion does not lie at the center of the {N_8_} cage, being closer to the pyridine groups and leaving space for further coordination with the electronegative anionic fluoride ligand. Overall, the structure is a distorted capped square antiprism (pseudo-*C*_4*v*_ symmetry). The short Ho–F^–^ bond [length = 2.129(4) Å] generates a pronounced axial CF,^40^ giving rise to a maximal *m*_*J*_ = ±8 projection (vide infra) associated with the quasi-doublet ground state (^5^I_8_) of the *J* = 8 (*L* = 6, *S* = 2) Ho^III^ ion. The complex crystallizes in the orthorhombic space group, *P*2_1_2_1_2 (Figure 1b), meaning that the unit cell comprises two molecules related by a 2-fold screw rotation. Accordingly, each molecule has a *C*_2_ axis passing through the Ho–F bond, which is reflected in four inequivalent Ho–N bond lengths [2.680(5), 2.522(6), 2.529(6), and 2.663(6) Å], as opposed to two. Although the point-group symmetry at each Ho^III^ site is strictly *C*_2_, departures from *C*_4_ are not discernible from these HFEPR studies [vide infra and the Supporting Information].

Like **1**, the Ho^III^ ion in **2** resides within a distorted square antiprismatic geometry imposed by the {N_8_} ligand cage, with an additional nitrogen from the neutral MeCN ligand replacing the axial F^–^ ligand of **1**. However, in contrast to **1**, compound **2** crystallizes in the monoclinic space group, *P*2_1_/*c*, with two inequivalent molecules in the asymmetric unit. The axial Ho–N (from MeCN) bond lengths are 2.499(10) Å (**2**^**a**^) and 2.542(8) Å (**2**^**b**^), which are close to the Ho–N distances of the {N_8_} ligand cage atoms [**2**^**a**^: Ho–N^cage^ = 2.516(9)–2.620(10) Å; **2**^**b**^: Ho–N^cage^ = 2.501(9)–2.601(9) Å]. The axiality of the CF is weaker for **2** in comparison to **1** due to both the neutrality of the MeCN ligand and because of the longer axial Ho–N bond when compared to the Ho–F^–^ distance; this results in low-lying CF states of mixed *m*_*J*_ = ±4 and ±3 character (vide infra), in contrast to the situation in **1**. Figure 1c–e depicts the two molecules in the asymmetric unit: as can be seen, not only are the pseudo-*C*_4_ axes of the {HoN_8_(MeCN)} molecular cores tilted with respect to each other by ∼48°, but the Ho–N–C–Me capping ligand is also relatively well aligned with the pseudo-*C*_4_ axis in one case (**2**^**a**^), whereas it is significantly bent in the other (**2**^**b**^). Consequently, one expects differently aligned zero-field splitting (ZFS) tensors for the two molecules as well as different overall ZFS parametrizations. In addition, there is some disorder in the structure of **2** that contributes to increased HFEPR line widths. As will be seen, this complicates the single-crystal studies of **2** because there is no unique magnetic symmetry axis and the resonances are broader in comparison to **1**. For example, a sharp eight-line pattern is observed in the spectra for **1** due to hyperfine coupling with the *I* = 7/2 ^165^Ho nucleus (100% natural abundance), whereas such fine structures are not resolved in the spectra for **2**.

### 3.1 HFEPR of Complex **1**

Figure 2 summarizes the results of a full angle dependence study of the 259 GHz, 2 K HFEPR spectrum of **1**. Analysis proceeds along the same lines as for the [Ho(W_5_O_18_)_2_]^9–^ system described in ref (34). At almost all orientations in Figure 2a, corresponding to rotation in a single azimuthal plane, ϕ = 70° (see inset), a spectrum with eight evenly spaced resonances is observed due to a single electronic transition, with the splitting caused by the hyperfine interaction with the *I* = 7/2 nuclear spin; indeed, each resonance can be associated with a particular nuclear *m*_*I*_ projection (, see Figure 2b). The spectrum exhibits a pronounced angle-dependence, indicative of a significant magnetic anisotropy. The positions of the eight resonances are plotted as a function of the polar angle, θ, in Figure 2b. The resonance positions diverge in the vicinity of θ = 0 and 180°, which is why spectra are only displayed from θ = 20 to 160°; outside of this domain, the resonances lie beyond of the maximum achievable vector field (4.5 T; see also the Supporting Information). The even spacing of the eight resonances enables a deconvolution of the electronic and hyperfine contributions at each field orientation:^34^ the pure electronic transition occurs at the center of the hyperfine pattern, which can be deduced by averaging the positions of the eight resonances. This procedure is then repeated for each plane of rotation, and the false color spherical plots in the inset of Figure 2b depict the electronic resonance field, *B*_res_, as a function of θ and ϕ, as viewed from two different perspectives. Smaller (larger) values of *B*_res_ correspond to easier (harder) magnetization directions, i.e., the resonance occurs at the lowest (highest) *B*_res_ in the regions where it is easiest (hardest) to Zeeman split the associated electronic levels. Thus, one can immediately visualize the easy-axis nature of the magnetic anisotropy, with the easy-axis at the dark-blue poles and the hard-plane at the dark-red equator. At first glance, the anisotropy also appears to be uniaxial, i.e., cylindrically, *C*_∞_, symmetric (the scarring seen in some regions near the equator is an artifact associated with the employed 2D mesh of θ and ϕ angles). Further fitting of *B*_res_(θ,ϕ) enables location of the easy-axis at approximately (θ_0_,ϕ_0_) = (92 ± 1°,76 ± 1°), i.e., close to the horizontal cavity end-plate on which the rod-shaped sample was mounted (inset to Figure 2a). Likewise, to within the experimental uncertainty, ϕ = 76° is aligned with the long edge of the crystal, i.e., the magnetic easy-axis (∥ *c*) is approximately aligned with the long axis of the crystal.

**Figure 3:**
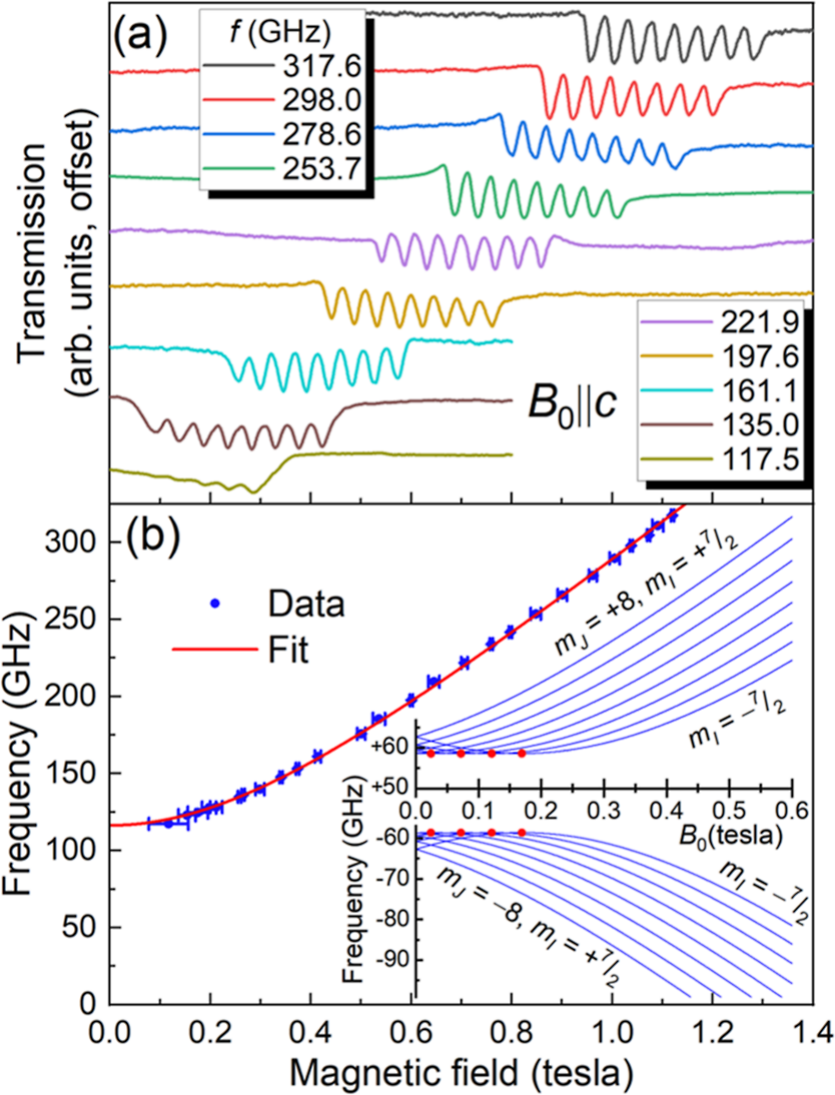
(a) HFEPR spectra of **1** recorded at 2 K as a function of frequency (see legend), with the applied field parallel to the easy- (*c*-) axis. (b) Frequency dependence of *B*_res_ determined from the spectra in (a) [along with many more that are not shown], with a best fit according to the Hamiltonian of eq 1 superimposed on the data (see text for definition of *B*_res_); the error bars reflect systematic uncertainties in the determination of *B*_res_ due to distortions in the resonance line shape. The inset shows a simulation of the hyperfine levels associated with the *m*_*J*_ = ±8 quasi-doublet, revealing the avoided level crossings, with 116.4 GHz SCTs indicated by red dots.

Having located the magnetic eas*y*-axis, a frequency-dependent study was performed with the magnetic field applied along this direction; the results are summarized in Figure 3. Magnetic measurements performed on the Dy^III^ analog of **1** demonstrated single-molecule magnet (SMM) behavior,^39^ which is attributed to the highly axial molecular structure. This results in an isolated ground doublet with a maximal *m*_*J*_ = ±15/2 projection and a large barrier to magnetization reversal. The Kramers nature of the ground state wave functions prevents their direct mixing, hence such species are nearly always EPR silent.^71^ A similar effect due to an axial CF is expected for the Ho^III^ complex (**1**) with an *m*_*J*_ = ±8 quasi-doublet ground state and a sizable separation to the *m*_*J*_ = ±7 excited states. However, a key difference in the Ho^III^ case is that it is not a Kramers ion, meaning that the ±*m*_*J*_ states can mix to the first order. In perfect *C*_4*v*_ symmetry, the allowed off-diagonal terms in a pure CF Hamiltonian are *B*_4_^±4^*Ô*_4_^±4^ and *B*_6_^±4^*Ô*_6_^±4^ (using the Stevens operator formalism for the *J* multiplet).^50^ The ground-state eigenvectors then comprise mixtures of the following *m*_*J*_ basis states: (+8,+4,0,–4,–8) & (−8,–4,0,+4,+8). Both functions have the same *m*_*J*_ composition but with different coefficients for the linear combinations: the leading terms having the largest coefficients, with smaller coefficients for successive *m*_*J*_ states. The main consequence arises at *B*_0_ = 0: the absence of CF terms that connect/mix the ground-state eigenvectors in the Dy^III^ (Kramers) case results in state energies that cross (exact degeneracy and absence of quantum tunneling), whereas in the Ho^III^ case, off-diagonal CF terms give rise to a degenerate perturbation and avoided level crossings, i.e., SCTs.^26^ The degenerate perturbation maximally mixes the (+8,+4,0,–4,–8) and (−8,–4,0,+4,+8) eigenvectors, giving rise to symmetric and antisymmetric combinations right at the SCT, for which parallel-mode (*B*_1_∥*B*_0_, where *B*_1_ is the microwave field) EPR transitions are allowed.^29^ Indeed, the fact that strong EPR spectra are observed is clear confirmation for the presence of the *B*_4_^±4^*Ô*_4_^±4^ and/or *B*_6_^±4^*Ô*_6_^±4^ interactions in the CF Hamiltonian of **1**.

The situation is slightly more complicated upon inclusion of hyperfine coupling. However, one may again reduce the problem to the pure electronic case by averaging the positions of the eight resonances (Figure 3a) and then plotting *B*_res_ versus frequency.^34^ As can be seen in Figure 3b, such a plot exhibits a finite gap of ≈116 GHz at zero magnetic field and a nonlinear dependence on *B*_0_ thereafter. This gap corresponds to the SCT frequency and is model-independent. One can further reduce the physics of a pure electronic ground-state quasi-doublet to that of an effective spin-1/2 subject to the following Hamiltonian
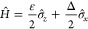
1where ε = *g*^eff^ μ_B_*B*_0_ denotes a diagonal Zeeman interaction, while the second term represents an off-diagonal interaction that causes an avoided crossing at *B*_0_ = 0, with a “tunneling” or SCT gap Δ; σ̂_*z*_ and σ̂_*x*_ represent the corresponding Pauli matrices, *g*^eff^ is the effective *g*-factor associated with the doublet and μ_B_ is the Bohr magneton. The eigen energies, *E*_±_, and EPR transition frequency, *f*, are then given, respectively, by  and , where *h* is Planck’s constant. The solid red curve in Figure 3b is a fit to the second expression, from which one can more precisely deduce Δ/*h* = 116.4 ± 1.0 GHz and *g*^eff^ = 18.97 ± 0.04. Such a large value of *g*^eff^ is anticipated for Ho^III^ with a dominant *m*_*J*_ = ±8 contribution to the ground-state doublet.^72^ Modifying the expression for the Zeeman interaction, taking into account the *m*_*J*_ composition of the ground-state eigenvectors, one obtains *g*^eff^μ_B_*B*_0_ ≡ *g*μ_B_*B*_0_δ*m*_*J*_, where δ*m*_*J*_ is the change in *m*_*J*_ associated with the EPR transition. Using the free-ion value for the Landé factor, *g*_*J*_ = 1.25,^72^ one obtains δ*m*_*J*_ = 15.2, which is close to the maximum of 16 expected for the pure |8,+8⟩ and |8,–8⟩ eigenvectors. The slight reduction from δ*m*_*J*_ = 16 is a clear indication of state mixing brought about by off-diagonal terms in the CF Hamiltonian [see eq 2 below], i.e., the ground-state eigenvectors contain admixtures of even *m*_*J*_ basis states, with |*m*_*J*_| < 8 (assuming rigorous *C*_2_ symmetry). One can then estimate the hyperfine coupling from the spacing between resonances in the eight-line spectrum (Figure 3a). In the high-field limit, this spacing is 46.8 ± 0.6 mT, which can be rescaled to give *A*/*h* = 775 ± 10 MHz; again, this is reduced from the expected free-ion value (810 mT)^72^ by about 4% due to state mixing.

Starting from the parameters estimated via the effective two-level description, we then attempt to simulate the results using the effective spin Hamiltonian of eq 2,^50^ taking into account the full Hilbert space associated with the *J* = 8 electron spin–orbital and *I* = 7/2 nuclear moments
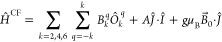
2where the double summation accounts for the CF interaction in terms of extended Stevens operators, *Ô*_*k*_^*q*^(*Ĵ*), of rank *k* and rotational order *q*, with associated *B*_*k*_^*q*^ coefficients;^73^ the second term parametrizes the electron–nuclear hyperfine interaction, with *Ĵ* and *Î* denoting the electron spin–orbital and nuclear momentum operators, respectively; while the last term represents the Zeeman interaction.

It is not possible to constrain the *B*_*k*_^*q*^ coefficients on the basis of the HFEPR study alone because the excited CF states are way beyond the frequencies employed even in the highest frequency EPR spectrometers.^74^ Therefore, we turn to CASSCF calculations (see below) to aid in the simulation. In the purely electrostatic case, approximate symmetry considerations limit the orders of the dominant CF terms to *q* = 0 and ±4, with *k* ≤ 6, i.e., the diagonal coefficients *B*_2_^0^, *B*_4_^0^ & *B*_6_^0^, and the off-diagonal ones, *Ô*_4_^±4^ & *Ô*_6_^±4^ [we note that the CASSCF calculations also give rise to weak *q* = 2 terms, reflecting the actual molecular *C*_2_ point group symmetry, but these do not produce any discernible effects on the simulations over the investigated field and frequency range (see the Supporting Information)]. Given the approximate agreement between the CASSCF calculations and experiment (vide infra), we employ the diagonal coefficients *B*_2_^0^, *B*_4_^0^ & *B*_6_^0^ for the purposes of simulation (see Table 1). This ensures a realistic separation of ∼100 cm^–1^ (≡ 3 THz, vide infra) between the *m*_*J*_ = ±8 quasi-doublet ground state and the lowest lying excited states. This gap is an order of magnitude larger than the ground-state Zeeman splitting in the field range of interest, as determined by the highest employed microwave frequency, *f* = 317 GHz, and is consistent with the absence of any experimental evidence for thermal population of excited CF states over the investigated temperature range (<10 K). Meanwhile, the similar forms of the *Ô*_4_^±4^ & *Ô*_6_^±4^ operators [respectively proportional to (*Ĵ*_+_^4^ ± *Ĵ*_–_^4^) and the symmetric product with (11*Ĵ*_*z*_^2^ – {*J*(*J* + 1) + 38})]^73^ make it impossible to determine their relative contributions to the SCT gap. Therefore, for the purposes of simulation, we restrict the adjustable parameters to a single off-diagonal CF term, *B*_4_^4^*Ô*_4_^4^, along with the values of *g* and *A*; all other parameters are set to zero.

**Table 1:** Parameters Obtained for **1** from a Combined HFEPR/CASSCF Analysis

parameter	value (frequency units)	value (cm^–1^)
*B*_2_^0^	–29.6 GHz^a^	–9.88 × 10^–^^1^
*B*_4_^0^	–68.7 MHz^a^	–2.29 × 10^–^^3^
*B*_6_^0^	–0.840 MHz^a^	–2.80 × 10^–^^5^
*B*_4_^4^	–1700 MHz^b^	–5.68 × 10^–^^2^
Δ	116.4 ± 1.0 GHz^c^	3.88 ± 0.03
*g*_*z*_	1.23 ± 0.01	
*A*_*z*_	800 ± 10 MHz	–2.67 × 10^–^^2^

a Constrained directly from CASSCF calculations—hence no associated uncertainty.

b Proxy for all allowed off-diagonal terms (*B*_4_^±4^ & *B*_6_^±4^).

c Only parameter that is not model-dependent.

The best simulation parameters are summarized in Table 1. It should be noted that the anisotropy observed in Figure 2b is governed by the axial CF interaction. This and the limited field range of the angle-dependent data do not permit constraining of the *x* and *y* components of *g̃* and *Ã*. Therefore, for the purposes of simulation, these are assumed to be isotropic [see eq 2]; in essence, the HFEPR study constrains only the *z*-components of the full *g̃* and *Ã* tensors (hence, only the *z*-components are given in Table 1). It should also be cautioned that there is an interdependence between *B*_4_^4^ and the diagonal (*q* = 0) CASSCF parameters because they all influence the *m*_*J*_ composition of the ground-state eigenvectors and, thus, affect the SCT gap, Δ. Nevertheless, use of the CASSCF results allows for a comparison between the *B*_4_^4^ parameter estimated for **1** and the well-studied [Ho(W_5_O_18_)_2_]^9–^ compound, for which *B*_4_^4^ = 3.14 × 10^–3^ cm^−1^,^26^ i.e., a factor ×18 smaller. Of course, the diagonal (*q* = 0) CF terms are also very different in the two cases, but the much larger *B*_4_^4^ value for **1** is consistent with an order of magnitude increase in the SCT frequency. There is also a weaker interdependency between *g*_*z*_, *A*_*z*_ and the CF parameters, meaning that the values given in Table 1 are somewhat dependent on the CASSCF results. However, as discussed previously,^42^ this interdependence diminishes as the ground-state doublet becomes more isolated. Consequently, the *g*_*z*_ and *A*_*z*_ values given in Table 1 should be quite reliable. Importantly, the values of *g*_*z*_ and *A*_*z*_ are very close to the expected free-ion values of 1.25 and 810 MHz, respectively;^72^ the <2% reduction may be indicative of weak covalency (see the Supporting Information for more detailed discussion).^75^

The parametrization in Table 1 gives rise to eight 116.4 ± 1.0 GHz SCTs, four on either side of zero-field, at positions *B*_0_^SCT^ = ±23.5, ±70.6, ±117.6, ±164.7 mT (see Figure 3b inset). These are remarkably similar to those determined for the [Ho(W_5_O_18_)_2_]^9–^ complex (±23.6, ±70.9, ±118.1, ±165.4 mT),^26^ indicating that the hyperfine interactions measured in magnetic field units are almost identical for the two compounds; the hyperfine parameters given in frequency units differ by about 4% (830 MHz for [Ho(W_5_O_18_)_2_]^9–^), potentially reflecting differences in covalency for the two compounds.^75^ It should be noted that the SCT positions, *B*_0_^SCT^, and the gap, Δ, are robust parameters obtained directly from this investigation without dependence on any model. The latter is more than an order of magnitude larger than the gap for the [Ho(W_5_O_18_)_2_]^9–^ complex. This has important consequences in terms of the second-order sensitivity to magnetic noise, where recent quantum dynamics simulations suggest that this could be limiting the coherence time, *T*_2_, at the SCT for the [Ho(W_5_O_18_)_2_]^9–^ molecule.^29^ The second-order sensitivity scales as d^2^*f*/d*B*_0_^2^ = γ_e_^2^/Δ, where γ_e_ is the electron gyromagnetic ratio, meaning that second-order sensitivity to magnetic noise is expected to be at least an order of magnitude weaker in compound **1**. Unfortunately, because of the large magnitude of the SCT gap, it is presently unfeasible to determine *T*_2_ for compound **1** due to the lack of any known pulsed EPR spectrometers operating exactly in the right frequency range.

Lastly, we return to the issue of local symmetry at each Ho^III^ site in **1**. There is no clear evidence for a 4-fold (or a 2-fold) symmetry from the false color plots in the inset to Figure 2b. This suggests that (a) the off-diagonal CF terms that give rise to the SCT have no discernible influence on the azimuthal (ϕ) angle-dependence of the HFEPR spectra below 4.5 T, in contrast to observations for several well-known SMMs,^47,48^ and (b) any *C*_2_ symmetric deviations in the Ho^III^ coordination environment away from exact *C*_4*v*_ point group symmetry also do not influence the spectra appreciably; we come back to this issue in discussing the CASSCF results (see below) where the *C*_2_ symmetry is clearly apparent. Closer scrutiny of the hyperfine splitting patterns at the most extreme polar angles (θ < 30° and θ > 150°) reveal more than eight resonances (see expanded view of θ = 20° spectrum in Figure 2a). Overall, the spectra at these extreme angles have the appearance of two overlapping eight-line patterns that are slightly shifted relative to each other. Hence, the resonances at the extremes have half the intensity (blue dots—Figure 2a) relative to those in the main part of the spectrum (red dots—Figure 2a). Moreover, there is a clear interference between the overlapping (incommensurate) hyperfine patterns, with resonances on the low-field side being well-resolved, whereas those on the high-field side are not. This could indicate two populations of molecules either having slightly different CF parameters or a weak azimuthal (ϕ-) variation in the polar angle- (θ-) dependence of the HFEPR spectra, an effect that would be most pronounced as θ approaches the hard- (*ab*-) plane. The time-reversal invariance of the spin–orbit interaction dictates that the two molecules in the unit cell that are related by the 2_1_ screw operation have identical spin-Hamiltonian parameters. Therefore, the only explanation for two or more populations of molecules would involve discrete disorder^47,48^ or crystal twinning in the *ab*-plane. We note, however, that while there are θ-dependent variations of the spectra close to the hard plane, there is no obvious two- or fourfold periodicity in ϕ.

### 3.2 HFEPR of Complex **2**

**Figure 4:**
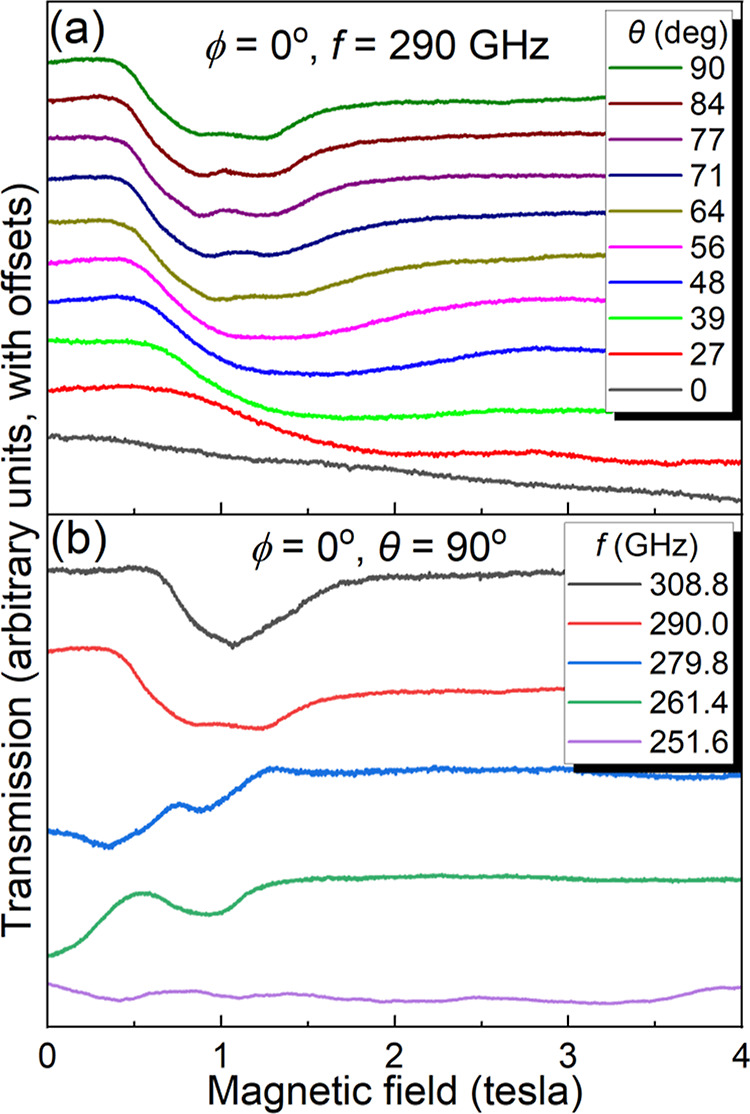
Angle- (a) and frequency- (b) dependent HFEPR spectra recorded at 2 K for compound **2**, with the same experimental geometry as depicted in the inset to Figure 2a; see legends for additional parameters.

Figure 4 summarizes both the angle- and frequency-dependent results. A broad angle-dependent feature is observed at a frequency of 290 GHz (Figure 4a). However, the characteristic eight-line ^165^Ho pattern is not observed. We presume that the hyperfine structures are not resolved due to disorder in this sample. In spite of this, the angle-dependent behavior strongly suggests that the HFEPR signal is due to an anisotropic Ho^III^ species, i.e., the resonance shifts with angle in a similar fashion to the data in Figure 2, occurring at ∼1 T at θ = 90° and moving to ∼2 T at θ = 27°. In fact, at the lowest fields, one can resolve two broad features that likely correspond to the two differently oriented molecular species within the unit cell of compound **2**. Unfortunately, it is not possible to separately follow the angle-dependence of these broad resonances, from which one could deduce the relative orientations of the corresponding species (**2**^**a**^ and **2**^**b**^). We therefore focus on the frequency dependence for the case where the resonance field is at a minimum (Figure 4b), which corresponds to reasonable alignment (to within <30°) of the applied magnetic field with the pseudo-4-fold axes of one or both species. For frequencies above 250 GHz, a pair of resonances is observed that move to higher field with increasing frequency. Below 250 GHz, the sensitivity of the spectrometer only improves, yet no evidence for EPR absorption is found (including for all of the frequencies employed in Figure 3a). This strongly suggests that the sample is EPR silent below this frequency; i.e., there is an ∼250 GHz SCT gap within the lowest-lying quasi-doublet (effectively a pair of singlets) associated with the *J* = 8 ground state of **2**.

Because of the limited frequency range over which resonances are observed for **2**, it is not possible to constrain the effective Landé factor, *g*^eff^, on the basis of eq 1. In turn, this means that it is not possible to determine the dominant *m*_*J*_ composition of the ground-state quasi-doublet. However, the clear tendency of the resonances to move toward zero-field with decreasing frequency, and the occurrence of a broad 261.4 GHz absorption right at zero-field, strongly suggests a SCT gap in the vicinity of 250 GHz. Therefore, these results demonstrate a systematic tuning of the SCT frequency in this family of compounds by varying the strength of the axial CF, thus supporting the original hypothesis. In this example, Ho^III^ coordination to neutral MeCN at the axial position is expected to result in a weaker CF compared to the negatively charged F^–^ ion of **1**,^76^ which also forms a shorter Ho–F bond. In turn, the weaker CF will result in reduced energy separations within the manifold of 2*J* + 1 = 17 *m*_*J*_ projection states, leading to greater admixing among the two lowest-lying basis states (see below for a greater insight), causing increased level repulsion and a pronounced increase in the SCT gap, Δ.

### 3.3 CASSCF Results

**Figure 5:**
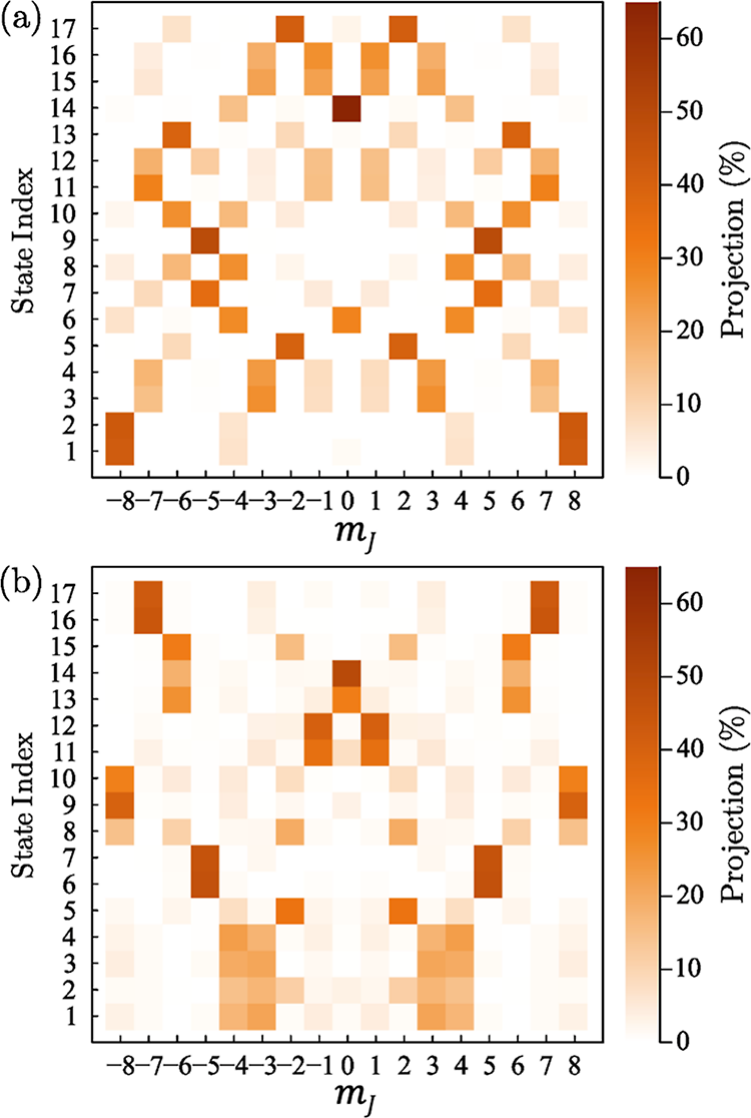
Relative contributions of the basis states of the *Ĵ*_*z*_ operator to the lowest 17 eigenstates of the Hamiltonian of eq 2, in zero magnetic field (neglecting the hyperfine interaction), for compounds **1** (a) and **2**^**b**^ (b) [see the Supporting Information for a comparison between **2**^**a**^ and **2**^**b**^]; *m*_*J*_ is the projection of *J* along the pseudo-*C*_4_ axis of the molecules. Note that the maximum projection of any basis state for time-reversed pairs (quasi-doublets) is 50%, hence the much stronger projection of *m*_*J*_ = 0 for state 14 for **1**, which is a singlet in nature.

**Figure 6:**
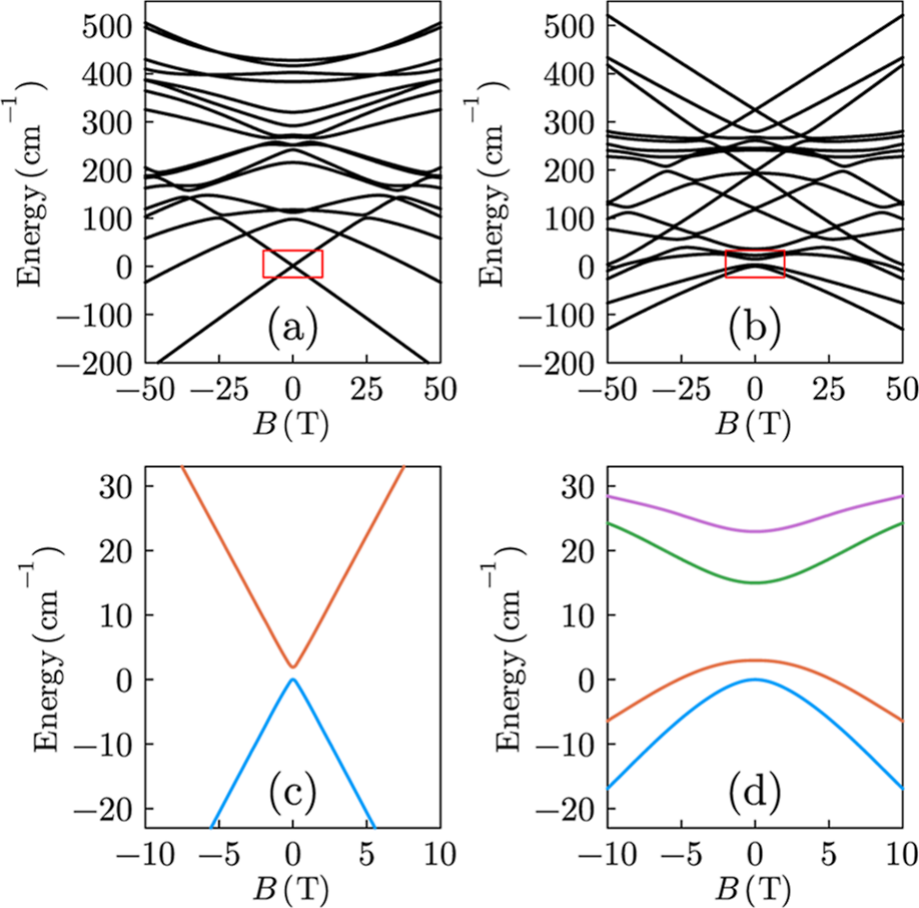
Zeeman energy level diagrams for compounds **1** (a) and **2**^**b**^ (b), with the applied magnetic field parallel to the local easy-axis, based on the spin Hamiltonian parametrized by the CASSCF calculations with DKH and dynamical correlation corrections (see the Supporting Information where comparisons are also made between **2**^**a**^ and **2**^**b**^). The low-lying levels and SCTs highlighted by the red squares in (a,b) are magnified in (c,d), respectively.

Figure 5 depicts the theoretical state compositions of the 17 lowest energy eigenvectors for compounds **1** and **2**^**b**^ under zero magnetic field (see Supporting Information Figure S1 for comparison between **2**^**a**^ and **2**^**b**^). The first thing to note is the very different compositions of the lowest-lying states for the two compounds (the state compositions for **2**^**a**^ and **2**^**b**^ are similar, see the Supporting Information): the lowest doublet for **1** is nearly a 50:50 mixture of |8,–8⟩ and |8,8⟩ (hereon, we denote such time-reversed pairs as |8,±*m*_*J*_⟩) with successively weaker admixtures of |8,±4⟩ and |8,0⟩; meanwhile, the lowest four levels of **2** (see also Figure 6) consist of almost equal mixtures of |8,±4⟩ and |8,±3⟩, with the |8,±8⟩ states lying much higher in energy. One may rationalize this behavior with reference to the aspherical Hund’s rule 4f^10^ charge density.^36^ The *m*_*J*_ = ±3, ±4, and ±5 states have similar shapes, with nodes oriented toward the magic angle, thereby naturally accommodating a square antiprismatic coordination. It is for this reason that these states lie lowest in energy for the [Ho(W_5_O_18_)_2_]^9–^ complex^37^ (also compound **2**). The *m*_*J*_ = ±8 states also possess a node close to the magic angle, albeit less pronounced. However, more importantly, these states possess a very sharp node at the axial position. For this reason, we hypothesize that the addition of the very strong anionic F^–^ ligand at the axial position in **1** stabilizes the *m*_*J*_ = ±8 ground states relative to all others. This reasoning can also be used to rationalize several other observations for compound **1**. For example, the *m*_*J*_ = ±7 charge densities possess a pronounced maximum at the axial position (as do *m*_*J*_ = 0, ±1, and ±6), explaining their location at much higher energies for **1**. Meanwhile, the *m*_*J*_ = ±3, ±4, ±5 states lie in order above the *m*_*J*_ = ±8 ground state for **1**, punctuated only by the lower half of the *m*_*J*_ = ±2 quasi-doublet, which is massively split due to the dominant transverse *Ô*_4_^4^ CF interaction, i.e., the center-of-mass of this pair lies much higher in energy. By contrast, replacing F^–^ with a much weaker neutral ligand (essentially a coordinating solvent) in **2** destabilizes the *m*_*J*_ = ±8 pair, favoring instead low-lying mixed *m*_*J*_ = ±3 and ±4 states, with ±5 just above. This illustrates the fact that it is the nodes in the charge density close to the magic angle that dominate the low-energy state ordering for compound **2** due to the absence of the strong anionic axial ligand. Meanwhile, states with maxima in their charge density at these angles (e.g., ±6 and ±7) lie much higher in energy.

Figure 6 displays Zeeman energy level diagrams (for *B*_0_ parallel to the pseudo-4-fold axes) corresponding to the full manifold of 17 eigenstates for compounds **1** and **2**^**b**^ (see Supporting Information Table S1 for zero-field eigenvalues and Figure S2 for comparisons between **2**^**a**^ and **2**^**b**^), including expanded views of the low energy SCTs. The *J* = 8 manifold spans 428 cm^–1^ for compound **1** and an average of 335 cm^–1^ for compound **2**. Again, the larger overall splitting in **1** is expected due to the stronger axial F^–^ ligand in comparison to the neutral coordinating acetonitrile molecule of **2**. As discussed above, a well isolated *m*_*J*_ = ±8 quasi-doublet ground state is found for **1**, with a theoretical SCT gap of 1.87 cm^–1^ (56.1 GHz). The situation in **2** is more complex (Figure 6d), with four low-lying mixed states and multiple clock-like transitions between them (an additional fifth state lies not far above). The lowest lying zero-field SCTs between states 1 (blue curve) and 2 (red curve) have gaps of 3.11 and 3.39 cm^–1^ (93.3 and 102 GHz), respectively, for **2**^**a**^ and **2**^**b**^ (see Supporting Information); we note that all of the theoretical SCTs occur exactly at *B*_0_ = 0, as the hyperfine interaction is not considered in the CASSCF calculations. The theoretical SCT gaps are 2× to 2.5× smaller than the experimental ones, most likely due to the perturbation treatment of the SOC interaction.^77^ However, the calculations support the hypothesis that replacing the axial F^–^ ligand with the much weaker acetonitrile ligand will lead to a reduction in the overall axial anisotropy and a corresponding increase in the SCT gap. The calculated increase in the SCT gap is in the range from 1.7× to 1.9× for **2**^**a**^ and **2**^**b**^, which compares favorably with the factor of 2.2× estimated from the experiments on **1** and **2**.

The pseudo-*C*_4*v*_ symmetry of **1** results in the *q* = ±4 CF terms dominating the state mixing (*B*_*k*_^*q*^ coefficients deduced from the CASSCF calculations are tabulated in the Supporting Information for both compounds—see Tables S2 and S3). This can be seen in Figure 5a, where the visible ground-state compositions are a mixture of the *m*_*J*_ = ±8, ±4, and 0 basis states. However, the calculations also generate weak *q* = ±2 and ±6 CF terms (generally 1–2 orders of magnitude smaller than the *q* = ±4 terms of same rank, for *q* ≤ 6), reflecting the actual *C*_2_ point group symmetry of **1**. Theoretical simulations of the spherical plots shown in the inset to Figure 2b do reveal evidence for the *C*_2_ and *C*_4*v*_ symmetries (Supporting Information Figure S3), albeit only above ∼15 T, i.e., the lack of evidence for these symmetries over the field range explored in the HFEPR investigations does not contradict the theoretical predictions. The absence of symmetry in **2** means that there are no restrictions on the allowed *B*_*k*_^*q*^ coefficients. The CASSCF calculations also predict CF terms with 6 < *q* ≤ 12 (Supporting Information Table S3). These interactions, which are forbidden for a purely electrostatic CF, do make small but measurable contributions to the eigenvalues, suggesting some degree of covalency.^75^

Finally, we note that minor variations in the structures of **2**^**a**^ and **2**^**b**^ result in very significant differences in the Zeeman splitting of the two lowest-lying eigenstates at magnetic fields below 5 T (see Supporting Information Figure S2): the lowest level exhibits marked zero-field curvature for **2**^**a**^ and less so for **2**^**b**^; meanwhile, for **2**^**a**^, the second level exhibits three turning points due to the closer approach of (and interaction with) the third level, whereas such behavior is less apparent for **2**^**b**^. These differences likely explain the complex double-peaked EPR spectra observed for compound **2** and probably also the much broader line widths in comparison to **1** on account of an extreme sensitivity to minor changes in structure, i.e., disorder, which is significant in **2**. Chemical tuning of the relative spacing between these low-lying states might lead to a situation in which the multiple turning points merge to a single point at *B*_0_ = 0, resulting in a vanishing of both the first and second derivatives, d*f*/d*B*_0_ and d^2^*f*/d*B*_0_^2^, leading to a second order SCT where both first and second order sensitivity to magnetic noise vanishes; indeed the situation in **2**^**b**^ appears to be very close to this limit. This could represent an additional strategy toward greatly enhanced coherence in molecular spin qubits.

## 4 Conclusions

We have carried out detailed single-crystal HFEPR investigations of the nine-coordinate lanthanide compounds [Ho^III^LF](CF_3_SO_3_)_2_·H_2_O (**1**) and [Ho^III^L(MeCN)](CF_3_SO_3_)_3_·0.5MeCN (**2**). The experimental results are augmented by detailed CASSCF calculations. The encapsulating nature of the octadentate ligand L, whereby it wraps around the lanthanide ion, precludes a rotoinversion axis, resulting in pseudo-*C*_4*v*_ symmetry for both compounds. In **1**, the ligation is completed by an electronegative fluoride ion at the apical position, resulting in a highly axial CF with an eas*y*-axis anisotropy and an *m*_*J*_ = ±8 quasi-doublet ground state that is well separated from excited CF states. More importantly, a significant off-diagonal (primarily tetragonal) CF interaction gives rise to a giant 116.4 ± 1.0 GHz SCT (avoided crossing) within this doublet, as determined by frequency-dependent HFEPR measurements. The CASSCF calculations reproduce the tetragonal CF interaction, with weaker rhombic terms reflecting the orthorhombic crystal structure of **1**; the predicted SCT gap (56.1 GHz) is within a factor of 2.1× of the experimental one. Although angle-dependent HFEPR studies are unable to detect either *C*_4_ or *C*_2_ symmetry-breaking interactions (the spectra are cylindrically symmetric), the theoretical results indicate that their effects should be observable only at much higher magnetic fields.

The apical coordination site occupied by the fluoride ion in **1** provides opportunities for tuning the strength of the axial CF. In turn, this influences the degree of state mixing within the ground quasi-doublet, which ultimately dictates the magnitude of the SCT gap. In this way, we demonstrate systematic engineering of the SCT frequency by varying the identity of the axial ligand. The negatively charged fluoride ion of **1** generates a strong axial CF in comparison to that of the nitrogen associated with the neutral MeCN ligand of **2**. In turn, the weaker axiality in **2** results in greater state mixing and an increase in the SCT frequency by more than a factor of 2 relative to **1**. This contrasts the situation for the only other Ho^III^ clock qubit that has been studied extensively to date,^17,26,29,34^ where the SCT gap is dictated by minor departures from ideal *D*_4*d*_ coordination geometry caused by unpredictable crystal packing forces associated with low-symmetry structure.

The rational design principles demonstrated in this study are highly desirable because second-order sensitivity to magnetic noise scales as γ_e_^2^/Δ, suggesting a means for further enhancing the low-temperature coherence of molecular clock qubits relative to those that have been studied up to now.^26,28,33^ Future work will aim to test this hypothesis by tuning the SCT frequency into ranges accessible with state-of-the-art pulsed HFEPR spectrometers, e.g., the 94 ± 0.5 GHz achievable with the HiPER spectrometer^78,79^ at the US National High Magnetic Field Laboratory. Finally, we note that the strategy of employing an encapsulating ligand with an open coordination site is likely to be more robust when molecules are deposited on surfaces or integrated into devices.

## Supporting Information Available

Further details of the computational studies, including tables with numerical eigenvalues, the computed *B*_*k*_^*q*^ coefficients and *g̃*-tensors for all compounds; comparisons between different levels of theory for **1**; and figures comparing **2**^**a**^ with **2**^**b**^ and calculations of the angle-dependent HFEPR spectra (PDF)
