# Supplementary material for: Engineering Clock Transitions in Molecular Lanthanide Complexes
Source: J Am Chem Soc. 2024 Apr 15;146(16):11083–94. doi: 10.1021/jacs.3c09353 (PMC11046435; doi:10.1021/jacs.3c09353)
Supplement: Supplementary file 1 — ja3c09353_si_001.pdf [file ja3c09353_si_001.pdf]

Supporting Information for: Engineering Clock Transitions in Molecular Lanthanide Complexes

Robert Stewart,[a,b,c] Angelos B. Canaj,*[,d,†] Shuanglong Liu,[c,e] Emma Regincós Martí,[d] Anna Celmina,[d] Gary Nichol,[f] Hai-Ping Cheng,[c,e] Mark Murrie*[,d] and Stephen Hill*[,a,b,c]

[a] National High Magnetic Field Laboratory, Florida State University, Tallahassee, FL 32310, USA

[b] Department of Physics, Florida State University, Tallahassee, FL 32306, USA

[c] Center for Molecular Magnetic Quantum Materials, University of Florida, Gainesville, FL 32611, USA

[d] School of Chemistry, University of Glasgow, University Avenue, Glasgow G12 8QQ, U.K.

[e] Department of Physics, Northeastern University, Boston, MA 02115, USA

[f] EastCHEM School of Chemistry, The University of Edinburgh, David Brewster Road, Edinburgh, EH9 3FJ, Scotland, U.K.

[†] Current address: The University of Edinburgh, School of Chemistry

*****Email: tsanai.angelos@gmail.com; mark.murrie@glasgow.ac.uk; shill@magnet.fsu.edu

Contents | Page

1. CASSCF Calculations **S2**

Table S1 – Theoretical Energy Eigenvalues **S4**

Figure S1 – Eigenstate Compositions for 2[a] and 2[b] **S5**

Table S2 – Theoretical CF Parameters **S6**

Table S3 – High Rank CF Parameters **S7**

Table S4 – Landé g-tensors **S9**

Figure S2 – Low Lying Energy Levels for 2 **S9**

Table S5 – DKH Correction and Dynamical Correlation **S10**

2. Theoretical Calculation of Angle Dependence of HFEPR Spectra **S11**

Figure S3 – Theoretical HFEPR Angle Dependence **S11**

3. References **S12**

1. CASSCF Calculations

The $4f^{10}$ Hund's rule ground state electronic configuration possesses orbital and spin angular momentum quantum numbers, $L = 6$ and $S = 2$, respectively, with maximal spin-orbital moment $J = L + S = 8$, and Landé factor $g_J = 1.25$. The Pauli principle prevents states with spin $S > 2$. To this end, the average energies of the lowest 21 $S = 2$ roots and the lowest 15 $S = 1$ roots were minimized during CASSCF calculations for both compounds **1** and **2**. These are the lowest 36 energy roots among all possible spin-allowed eigenstates; their weights were set equal to each other. The lowest $S = 0$ root is 34,000 cm^{-1} (4.2 eV) above the ground state, i.e., the energies of $S = 0$ roots are so high that we did not include any of them in the state-average CASSCF calculations. For both **1** and **2**, the Ho 4f orbital contribution to the active molecular orbitals is more than 99% after self-consistency. Meanwhile, the Ho 4f orbital contribution is less than 14% for the unoccupied molecular orbitals lower than 260 eV relative to the highest (partially) occupied orbital. This suggests that the size of the active space is likely sufficient.

Table S1 gives the energies of the lowest 17 eigenstates for the lone structure of **1** and the two distinct structures of **2**; see Fig. 1 in main text for geometries of **1**, $\mathbf{2}^{a}$ and $\mathbf{2}^{b}$. The next excited state, i.e., the 18th eigenstate, is 5,130 cm^{-1} (0.64 eV) above the ground state for both compounds. Consequently, the ground state $J = 8$ manifold is easy to identify due to its large energy separation from the next manifold of excited states. The $J = 8$ manifold spans 428 cm^{-1} for **1**, and an average value of ~335 cm^{-1} for **2**, confirming the reduced axial crystal field (CF) for the latter. In the case of compound **2**, the energy span of the $J = 8$ states is reduced by about 19 cm^{-1} when the acetonitrile group tilts away from the *pseudo*-C_4 symmetry axis ($\mathbf{2}^{\mathbf{b}}$). This distortion also enhances the mixing of the four lowest energy eigenstates relative to $\mathbf{2}^{\mathbf{a}}$, as can be seen from the fact that the weights of $|8,-4\rangle$, $|8,-3\rangle$, $|8,3\rangle$, and $|8,4\rangle$ become closer to each other (see Fig. S1).

In order to provide an estimate of numerical accuracy, calculations were performed for mirror images of molecule $\mathbf{2}^{\mathbf{a}}$ within the unit cell of compound **2**. These calculations should yield identical energy eigenvalues; however, the obtained results exhibit small differences. Therefore, the values for $\mathbf{2}^{\mathbf{a}}$ in Table S1 represent an average for the mirror images, while the deviation from the average is provided as a numerical error; similar errors are anticipated for **1** and $\mathbf{2}^{\mathbf{b}}$.

Theoretical CF parameters, B_k^q, associated with the extended Stevens operators, $\hat{O}_k^q(\hat{J})$,[1] of rank k and rotational order q, are given in Tables S2 & S3. These parameters, along with the corresponding *g*-tensor components given in Table S4, were estimated according to the methods outlined in Ref. [2], exploiting the projection properties of irreducible tensor operators. Given the apparent errors inherent in the numerical accuracy of the CASSCF calculations (see above), it is natural that small errors propagate through to the computed CF parameters. Indeed, finite values are found for all B_k^q coefficients, including those of rank $k > 6$ (Table S3). As a first check, we set all odd q coefficients for compound **1** equal to zero, as these are unphysical due its C_2 symmetric structure; these are of the order 10^{-6} cm^{-1} for $k = 2$, 10^{-8} cm^{-1} for $k = 4$, $< 10^{-9}$ cm^{-1} for $k = 6$, and $< 10^{-11}$ cm^{-1} for $k > 6$, contributing insignificantly to the energy eigenvalues and spin clock transition (SCT) gap. Consequently, their removal apparently does not significantly affect the zero-field energy eigenvalues computed via Eq. (2) (neglecting hyperfine interaction) in the main article. These coefficients are larger for compound **2** due to its low symmetry structure, hence they are retained in Tables S2 and S3. B_k^q coefficients of rank $k > 6$ are unphysical in a purely electrostatic CF model. However, they may become significant when there is some degree of covalency.[3] Removing them has a measurable effect on the energy eigenvalues, particularly for the higher-lying states (of up to ~10 cm^{-1} for **1** and ~20 cm^{-1} for **2**). We therefore tabulate these coefficients separately in Table S3, noting that their omission will lead to differences in the energies computed via the Stevens formalism when compared to those in Table S1.

Remarkably, as seen in Fig. S2, the minor variations in the structures of $\mathbf{2^a}$ and $\mathbf{2^b}$ (along with the associated spin Hamiltonian parameters in Tables S2 to S4) result in very significant differences in the Zeeman splitting of the two lowest-lying eigenstates at low magnetic fields (< 5 T): the lowest level exhibits marked curvature for $\mathbf{2}^a$ and less so for $\mathbf{2}^b$; meanwhile, for $\mathbf{2^a}$, the 2nd level exhibits three turning points due to the closer approach of (and interaction with) the third level, whereas such behavior is less apparent for $\mathbf{2^b}$. These differences likely explain the complex double-peaked EPR spectra observed for compound **2**, and probably also the much broader linewidths in comparison to **1** on account of an extreme sensitivity to minor changes in structure, i.e., disorder. Interestingly, chemical tuning of the relative spacing between these low-lying states might

lead to a situation in which the multiple turning points merge to a single point at $B_0 = 0$, resulting in a vanishing of both the first and second derivatives, df/dB_0 and d^2f/dB_0^2 (where f is the clock frequency), leading to a 2nd order SCT where both first and second order sensitivity to magnetic noise vanishes. This could represent an additional strategy towards greatly enhanced coherence in molecular spin qubits.

Finally, Table S5 illustrates the effects of the 2nd order Douglas–Kroll–Hess (DKH) correction and dynamical correlation (2nd order N-electron valence state perturbation theory, NEVPT2) for **1**. The former has a marginal influence on the eigenvalues; the maximal change in the lowest 17 eigenvalues is 0.7%. By contrast, dynamical correlation gives rise to more appreciable corrections to the eigenvalues, e.g., the SCT gap is enhanced by 28% and the span of the $J = 8$ manifold increases by 16%.

Table S1 – Theoretical Energy Eigenvalues. Theoretical values of the lowest 17 energy eigenvalues associated with the $J = 8$ ground-state manifold for compounds **1** and **2**. These calculations include the DKH correction and dynamical correlation.

State index	**1** (cm^{-1})	**2**[a] (cm^{-1})	**2**[b] (cm^{-1})
1	0.00	0.00	0.00
2	1.87	3.11 ± 0.38	3.39
3	97.88	10.70 ± 0.04	14.47
4	112.05	15.72 ± 0.07	23.37
5	117.10	28.68 ± 0.42	36.25
6	215.61	114.45 ± 0.05	117.15
7	241.95	120.43 ± 0.01	121.10
8	251.21	196.89 ± 0.02	191.51
9	251.96	223.56 ± 0.22	195.39
10	268.22	228.30 ± 0.15	198.96
11	272.31	244.68 ± 0.06	241.49
12	291.60	252.90 ± 0.81	244.86
13	319.90	269.54 ± 0.03	261.10
14	383.28	272.86 ± 0.65	268.37
15	402.07	289.47 ± 0.02	280.07
16	416.23	343.52 ± 0.05	324.08
17	427.64	344.83 ± 0.10	325.55

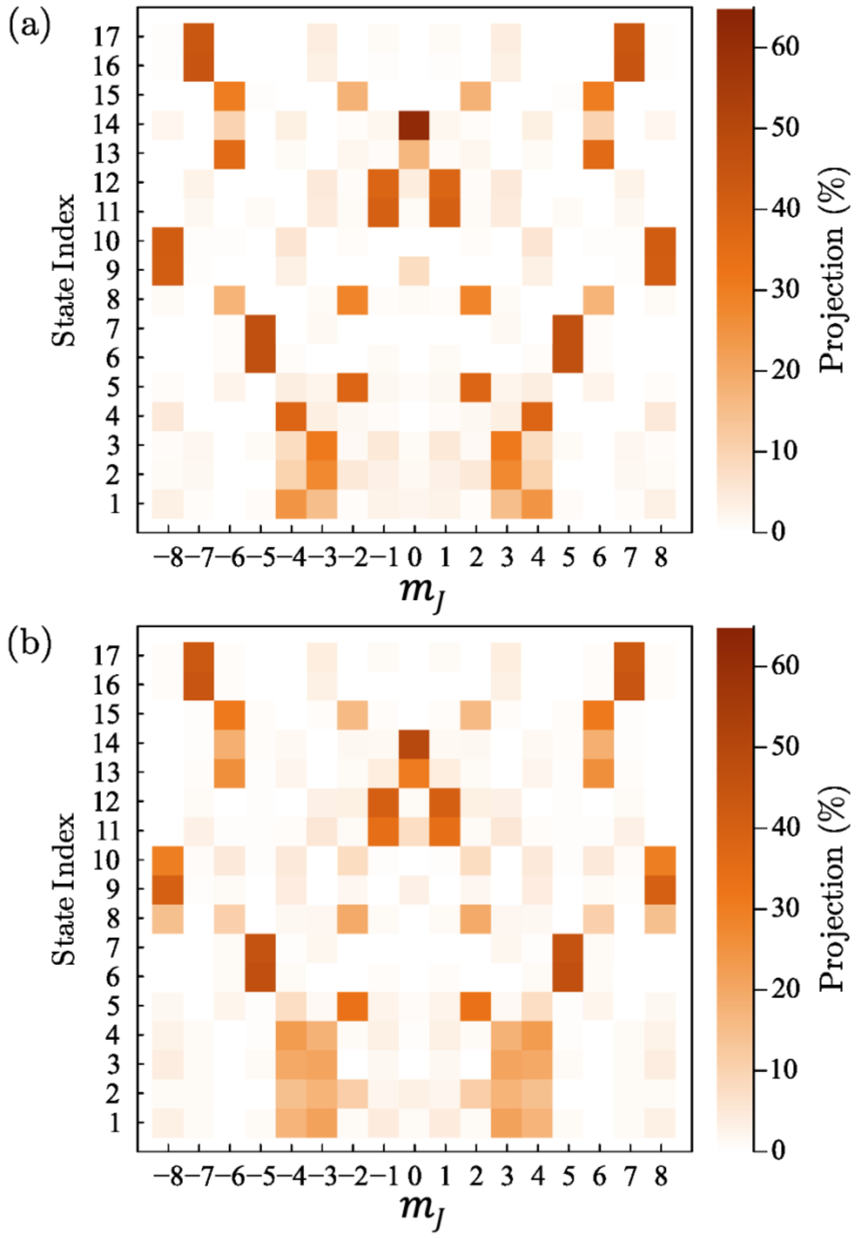

Figure S1 – Eigenstate Compositions for 2[a] and 2[b]. Compositions of the lowest 17 eigenstates of **2**[a] **(a)** and **2**[b] **(b)**; m_J is the projection of the total angular momentum along the *pseudo*-fourfold rotational symmetry axis of the molecule.

Table S2 – Theoretical CF Parameters. B_k^q values of rank $k \leq 6$ associated with extended Stevens operators, $\hat{O}_k^q$, for **1** and **2**;[1] the DKH correction and dynamical correlation were included in the calculations. Diagonal ($q = 0$) terms are highlighted by blue shading. Terms with $k > 6$ are given in Table S3 for both compounds. The principal axes of the magnetic tensors are tilted 2.6^o and 2.2^o relative to the *pseudo*-C_4 axes defined by the axial Ho—N bonds for **2**[a] and **2**[b], respectively.

k	q	**1** (cm^{-1})	**2**[a] (cm^{-1})	**2**[b] (cm^{-1})
2	-2	-1.439092E-02	+2.443228E-03	+2.814764E-03
2	-1	-	+1.762620E-02	+4.801920E-02
2	0	-9.879799E-01	+6.416838E-01	+5.067858E-01
2	1	-	+9.324418E-02	+3.521530E-02
2	2	+2.565350E-01	-2.058489E-01	-2.819945E-01
4	-4	+1.099533E-02	+1.003275E-02	+4.638045E-03
4	-3	-	+1.370394E-03	+2.656154E-03
4	-2	-6.791390E-04	-3.501028E-04	+4.253908E-04
4	-1	-	-9.692556E-04	-1.286340E-03
4	0	-2.290932E-03	+1.168395E-03	+7.479891E-04
4	1	-	-8.005073E-04	-1.236961E-03
4	2	+6.268229E-04	-6.055324E-04	+4.268900E-04
4	3	-	+2.721276E-03	-3.918420E-03
4	4	+1.234774E-02	-7.157236E-03	-1.099096E-02
6	-6	-4.297295E-05	-7.387451E-06	+1.073399E-06
6	-5	-	+7.388847E-05	-2.241612E-04
6	-4	-5.133753E-04	-4.011000E-04	+1.088062E-04
6	-3	-	-1.268996E-05	-7.007113E-05
6	-2	+1.069108E-05	-2.023537E-05	-7.741082E-06
6	-1	-	+8.060693E-05	+1.123947E-04
6	0	-2.801651E-05	-5.457237E-05	-5.294558E-05
6	1	-	+3.348331E-05	+3.277374E-05
6	2	-6.685182E-06	+3.008178E-05	+2.484965E-05
6	3	-	-8.113858E-05	+6.716592E-05
6	4	-1.283669E-04	-1.706615E-05	+3.504876E-04
6	5	-	-1.393847E-04	+3.207024E-05
6	6	-3.273081E-05	+2.412707E-05	-2.205788E-05

Table S3 – High Rank CF Parameters. B_k^q values of rank $k > 6$ associated with extended Stevens operators, $\hat{O}_k^q$, for **1** and **2**;[1] the DKH correction and dynamical correlation were included in the calculations. Diagonal ($q = 0$) terms are highlighted by blue shading. These terms, which are unphysical in a purely electrostatic picture, may arise due to covalency effects.[3]

k	q	**1** (cm^{-1})	**2**[a] (cm^{-1})	**2**[b] (cm^{-1})
8	-8	+7.180513E-07	-1.066012E-08	+3.288885E-07
8	-7	-	+1.502806E-07	-1.880668E-07
8	-6	+9.196982E-08	+9.353980E-08	-3.756519E-08
8	-5	-	+3.723723E-07	-1.578078E-07
8	-4	-8.176609E-08	-1.189865E-07	+9.776474E-08
8	-3	-	+5.363510E-08	+8.615388E-08
8	-2	+2.149869E-08	-4.572259E-09	+2.431305E-08
8	-1	-	-5.038066E-08	-7.598247E-08
8	0	+1.102182E-08	+2.150728E-08	+2.208362E-08
8	1	-	-1.804177E-08	-1.778874E-08
8	2	-8.042906E-08	-1.831274E-08	-3.765462E-08
8	3	-	-1.247662E-08	-3.767980E-08
8	4	+1.425502E-07	-1.000640E-07	+1.983551E-09
8	5	-	-1.325889E-07	+5.789547E-08
8	6	+2.470700E-08	-8.768105E-08	-4.023042E-08
8	7	-	-2.715743E-09	-1.800215E-08
8	8	-8.998576E-07	-6.263946E-07	+5.347033E-07
10	-10	+1.097870E-10	+2.414560E-10	-1.651225E-10
10	-9	-	+4.095543E-09	-8.867020E-09
10	-8	+3.120414E-09	+1.566070E-10	+3.828651E-09
10	-7	-	+2.696575E-09	-2.851387E-09
10	-6	-3.250191E-10	+1.683074E-10	-5.314313E-11
10	-5	-	+3.406531E-10	-5.672803E-10
10	-4	+5.472887E-10	-5.369762E-09	+9.388733E-10
10	-3	-	-4.568644E-10	-1.470234E-09
10	-2	+9.833439E-11	+1.105969E-10	-3.280331E-11
10	-1	-	-1.087755E-10	-1.537052E-10
10	0	+3.822158E-11	+3.411946E-11	+3.058744E-11
10	1	-	-3.365831E-11	-5.612876E-12
10	2	+1.195656E-10	-1.144742E-11	-2.140593E-11
10	3	-	-1.811812E-09	+1.851962E-09

10	4	-1.602858E-09	-1.605912E-10	+3.592661E-09
10	5	-	-3.209799E-10	+2.117412E-10
10	6	+7.772361E-11	-1.584926E-10	+2.506288E-10
10	7	-	-2.534762E-10	+2.949420E-09
10	8	-6.363934E-09	-6.291754E-09	+6.496007E-09
10	9	-	+2.013364E-09	+1.980846E-09
10	10	+3.919224E-10	+7.075155E-10	-1.067351E-10
12	-12	-5.225112E-11	+2.426773E-11	+2.103512E-11
12	-11	-	-2.307667E-12	-4.619804E-12
12	-10	+2.595881E-12	-1.040130E-12	-2.873485E-12
12	-9	-	+5.664049E-12	-2.017542E-11
12	-8	-8.289323E-13	+3.051037E-13	+9.109504E-12
12	-7	-	+4.161113E-12	-2.447897E-12
12	-6	-8.195340E-13	+4.957993E-13	+5.374026E-13
12	-5	-	-6.606009E-13	-5.567837E-12
12	-4	-2.041798E-12	+3.161212E-12	-1.165784E-12
12	-3	-	+1.238020E-12	+2.647575E-12
12	-2	+1.237098E-12	-8.315495E-13	+2.190141E-13
12	-1	-	-9.334579E-13	-1.267993E-12
12	0	+1.065900E-13	+1.195263E-13	+1.060137E-13
12	1	-	-5.107802E-13	-4.855332E-13
12	2	+1.590444E-12	-2.898634E-12	-3.306676E-12
12	3	-	+2.252335E-12	-1.698031E-12
12	4	-3.344009E-12	+4.406514E-13	-1.650396E-12
12	5	-	-7.055753E-12	-2.661123E-12
12	6	+1.250856E-12	-1.704957E-13	-1.319624E-12
12	7	-	-2.353790E-12	+2.696641E-12
12	8	+1.880036E-11	-1.633972E-11	+1.188936E-11
12	9	-	+7.173566E-12	+6.418929E-12
12	10	-3.353270E-12	+7.575769E-12	+1.317469E-12
12	11	-	+1.131707E-11	+1.276924E-11
12	12	-5.245593E-11	+4.357842E-12	+1.465189E-11

Table S4 – Landé g-tensors. Theoretical values of the tensor components for the total $J = 8$ spin-orbital ground states of **1** and **2** (not to be confused with effective g-factors associated with the ground state quasi-doublet). The g-tensors are nearly isotropic for both compounds, and the g_z value for **1** is in good agreement with the experimental one.

	**1**	**2**[a]	**2**[b]
g_x	1.239	1.239	1.239
g_y	1.239	1.239	1.239
g_z	1.238	1.240	1.240

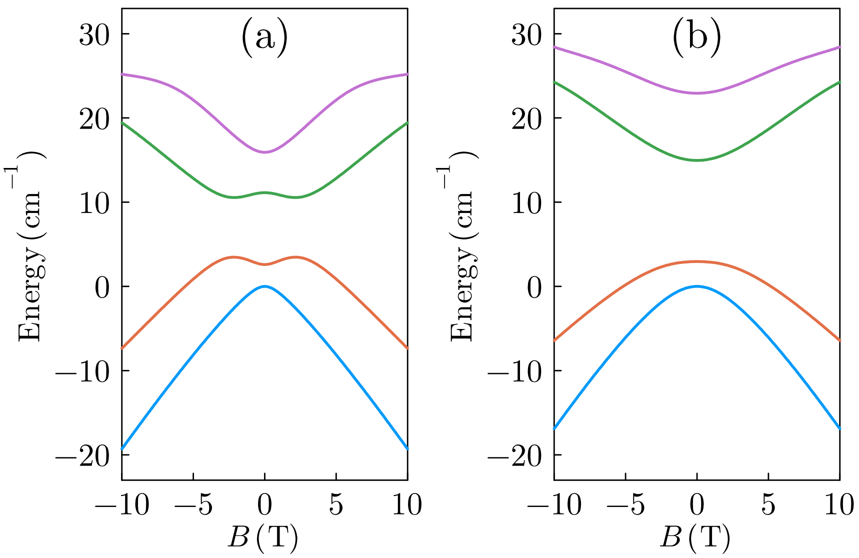

Figure S2 – Low Lying Energy Levels for 2. Zeeman diagrams for the four lowest energy levels of **2**[a] **(a)** and **2**[b] **(b)**.

Table S5 – DKH Correction and Dynamical Correlation. Low energy levels for **1** as calculated at different levels of theory.

CASSCF + SOMF (cm^{-1})	CASSCF + SOMF + 2nd order DKH correction (cm^{-1})	CASSCF + NEVPT2 + SOMF + 2nd order DKH correction (cm^{-1})
0.00	0.00	0.00
1.47	1.46	1.87
98.71	98.97	97.88
108.46	108.72	112.05
118.76	119.10	117.10
182.06	182.50	215.61
192.80	193.18	241.95
193.58	193.93	251.21
198.25	198.63	251.96
218.77	219.16	268.22
223.03	223.46	272.31
240.07	240.55	291.60
246.91	247.39	319.90
324.08	324.68	383.28
340.76	341.36	402.07
360.69	361.33	416.23
367.73	368.38	427.64

2. Theoretical Calculation of Angle Dependence of HFEPR Spectra

As seen from Table S2, the CASSCF calculations for compound **1** generate significant off-diagonal CF terms (with $k > 0$) that are expected to give rise to both a fourfold ($q = 4$) and twofold ($q = 2$) angle-dependence of the HFEPR spectra about the *pseudo*-C_4 molecular (crystallographic *c*-) axis. However, no such behavior is evident from the results presented in Fig. 2 of the main article. We demonstrate here that this is due to the limited field range of the vector magnet employed in these investigations, and that these effects would be observable at much higher fields (significantly above the range where such precision angle-dependent HFEPR measurements are currently feasible).

Figure S3 displays spherical false color plots of the pure electronic resonance position, $B_{\mathrm{res}}(\psi, \chi)$, generated using the CASSCF CF parameters given in Table S2 for compound **1**. We employ a different coordinate frame here, where ψ and χ represent polar and azimuthal spherical coordinates, with reference to the crystallographic frame, i.e., ψ is referenced to the crystal *c*-axis and χ denotes the plane of ψ rotation within the *ab*-plane. This transformation is necessary in order to distinguish from the lab coordinates employed in discussion of Fig. 2 in the main article.

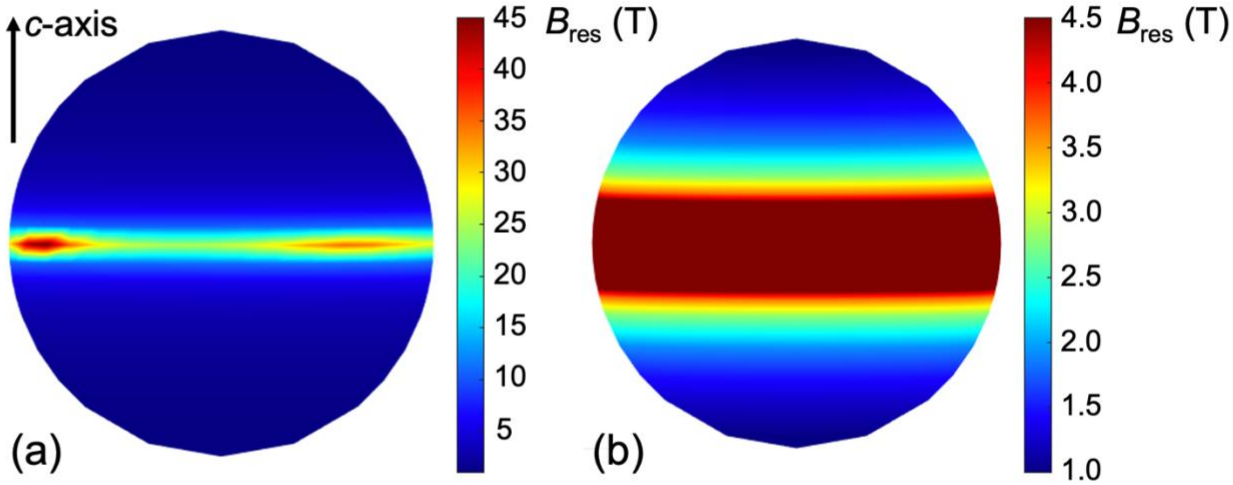

Figure S3 – Theoretical HFEPR Angle Dependence. Spherical false color plots of the pure electronic resonance position, $B_{\mathrm{res}}(\psi, \chi)$, truncated at 45 T **(a)** and 4.5 T **(b)**, viewed along a direction parallel to the crystallographic *ab*-plane.

As seen in Fig. S3(a), a very clear azimuthal (χ) dependence of the resonance position can be observed as the applied field approaches the hard (*ab*-) plane ($\psi = 90^{\mathrm{o}}$). The overall behavior indicates a superposition of both twofold and fourfold patterns, as

the two regions where B_{res} diverges the most (red color) exhibit different intensities; as viewed over one hemisphere, these regions are separated by ~90 degrees, with similar patterns on the opposite hemispheres (related by inversion through the center of the sphere), i.e., the red patches are separated by ~90 degrees (fourfold behavior) in the *ab*-plane, but their intensities alternate with a ~180 degree periodicity (twofold behavior). The angle-dependence is just about visible in the lighter blue regions in Fig. S3(a). However, these still correspond to fields above 15 T, which are not accessible with the employed vector magnet. For comparison, Fig. S3(b) displays the same data truncated at 4.5 T, which is the maximum vector field available for these experiments. As can be seen, there is absolutely no hint of any azimuthal angle-dependence at these low fields. Therefore, even though the HFEPR results in Fig. 2 of the main article exhibit cylindrical symmetry, they are nonetheless consistent with the CASSCF results and the underlying C_2 symmetry of the structure. Obviously, it would be desirable to perform high-field angle-dependent measurements in order to provide tighter constraints on the CASSCF parameterization. The simulations indicate azimuthal (χ-dependent) shifts in B_{res} of ~0.35 T at $\psi = 85^{\mathrm{o}}$ and in the 10.75 to 11.1 T range, which would be easily detectable if the required magnet were available; at $\psi = 80^{\mathrm{o}}$, the shifts are an order of magnitude weaker, where the resonance occurs at ~5.45 T. It should also be noted that these measurements are greatly complicated because the spectrum broadens as $\psi \rightarrow 90^{\mathrm{o}}$.

3. References

1 S. A. Altshuler, B. M. Kozyrev, Electron Paramagnetic Resonance in Compounds of Transition Elements, 2nd edn., Wiley (1974).

2 L. F. Chibotaru, L. Ungur, Ab initio calculation of anisotropic magnetic properties of complexes. I. Unique definition of pseudospin Hamiltonians and their derivation, J. Chem. Phys. **137**, 064112 (2012). https://doi.org/10.1063/1.4739763

3 A. S. Manvell, R. Pfleger, N. A. Bonde, M. Briganti, C. A. Mattei, T. B. Nannestad, H. Weihe, A. K. Powell, J. Ollivier, J. Bendix, M. Perfetti, LnDOTA puppeteering: removing the water molecule and imposing tetragonal symmetry, Chem. Sci. **15**, 113 – 123 (2024). https://doi.org/10.1039/D3SC03928E
